# Effects of Workout and Meditation Phenomenon Program on Body Composition, Flexibility, and Blood Pressure Data Analysis

**Published:** 2017-07

**Authors:** Eun-Ju CHOI, Wi-Young SO, Taikyeong Ted. JEONG

**Affiliations:** 1. Division of Sports Sciences, Konkuk University, Chungju, Korea; 2. Sports and Health Care Major, Korea National University of Transportation, Chungju, Korea; 3. Dept. of Computer Science and Engineering, Seoul Women’s University, Seoul, Korea

## Dear Editor-in-Chief

Overall, 3.4 million deaths, loss of 3.9% of life years, and 3.8% of disability-adjusted life-years were estimated to be caused by overweight and obesity worldwide in 2010 (1). Moreover, the worldwide proportion of adults aged over 20 yr with a body-mass index (BMI) of 25 kg/m^2^ or greater has increased between 1980 and 2013, from 28.8% (95% uncertainty interval 28.4–29.3) to 36.9% (36.3–37.4) in men and from 29.8% (29.3–30.2) to 38.0% (37.5–38.5) in women (1). Obesity has become a major global health problem owing to the established health risks associated with it and their substantial increases in prevalence.

Previous studies recommended encouraging regular exercise training for preventing chronic diseases such as obesity, hypertension, and diabetes (2–3). Regular exercise training such as dance and yoga programs is very popular and effective for preventing and treating chronic disease (4–5). However, only a few studies have investigated the effectiveness of dance and yoga programs (6). Therefore, this study aimed to examine the effects of dance and yoga programs on body composition, flexibility, and blood pressure in Korean college students.

A total of 161 college students in Chungju in 2016, to undergo measurements of body composition, flexibility, and blood pressure were included in the study. They were classified into a dance group (male=54, female=58; age, 22.96±1.93 yr; height, 169.10±8.54 cm; weight, 63.53±11.80 kg) and a yoga group (male=10, female=39; age, 22.37±1.76 yr; height, 166.06±7.41 cm; weight, 59.55±12.45 kg). All of them provided written informed consent to participate in this study.

The participants underwent body composition, flexibility, and blood pressure measurements before and after the intervention. The exercise groups participated in a dance and yoga program for 12 wk, respectively. The dance group participated in a dance program for 2 h once a week, for 12 wk, modified based on exercise (7). The yoga group participated in a yoga program for 2 h once a week, for 12 wk, modified based on the exercise protocol (8).

The muscle mass, percent body fat, visceral fat area, and basal metabolic rate were measured using Inbody 720 equipment (Biospace, Seoul, Korea). Flexibility (sit-and-reach) and resting heart rate were measured based on the book recommendations (9). Blood pressure was measured 3 times at 2-min intervals. The nurse specialist then determined the mean blood pressure for each subject.

All data are presented as mean±standard deviation. Paired t-tests were used to analyze differences between the dependent variables before and after the dance and yoga program, respectively. All analyses were performed using SPSS ver. 18.0 (SPSS, Chicago, IL, USA). Statistical significance was set at *P*<0.05.

After the dance program, a significant improvement was seen in only the sit-and-reach movement (*P*<0.001) compared to that before the program. While after the yoga program, significant improvements were seen in both the sit-and-reach movement (*P*<0.001) and systolic blood pressure (*P*=0.003) (Table 1). Thus, 12-wk participation in the dance or yoga program might not be effective in improving body composition and blood pressure, except flexibility, in the Korean college students.

**Table 1:** Changes in body composition, flexibility, and blood pressure after 12 wk of the dance (n=112) and yoga (n=49) program

**Variables**	**Group**	**Pre**	**Post**	**t**	***P***
Body mass index (kg/m^2^)	Dance	22.07±2.78	22.03±2.81	0.624	0.534
	Yoga	21.48±3.66	21.58±3.51	−0.326	0.746
Muscle mass (kg)	Dance	45.44±9.02	45.76±8.84	−1.149	0.253
	Yoga	41.52±7.62	41.61±7.76	−0.658	0.514
Body fat (%)	Dance	22.04±5.81	21.82±6.14	0.952	0.343
	Yoga	24.14±5.88	24.17±5.98	−0.129	0.898
Visceral fat area (cm^2^)	Dance	49.28±21.71	48.25±21.23	1.424	0.157
	Yoga	43.20±20.67	43.33±22.01	−0.167	0.868
Basal metabolic rate (kcal)	Dance	1417.40±188.28	1418.88±188.06	−0.785	0.434
	Yoga	1324.14±154.55	1326.08±159.18	−0.775	0.442
Sit-and-reach (cm)	Dance	12.12±8.35	15.15±8.64	−6.629	<0.001***
	Yoga	11.18±8.23	17.92±7.07	−9.551	<0.001***
Systolic blood pressure (mmHg)	Dance	123.78±14.61	119.47±13.65	3.001	0.003**
	Yoga	116.59±11.37	117.10±12.23	−0.522	0.604
Diastolic blood pressure (mmHg)	Dance	75.43±12.67	73.19±9.13	1.924	0.057
	Yoga	70.12±8.17	70.69±8.62	−0.317	0.753
Resting heart rate (beats/min)	Dance	80.90±13.12	81.46±12.30	−0.500	0.618
	Yoga	77.88±9.88	80.31±10.23	−1.776	0.082

Data are presented as means±standard deviations

** *P*<0.01,

*** *P*<0.001; tested by paired *t*-tests

## References

[1] Ng M Fleming T Robinson M (2014). Global, regional, and national prevalence of overweight and obesity in children and adults during 1980–2013: a systematic analysis for the Global Burden of Disease Study 2013. Lancet, 384(9945):766–81. 24880830 10.1016/S0140-6736(14)60460-8 PMC4624264

[2] Ambrose KR Golightly YM (2015). Physical exercise as non-pharmacological treatment of chronic pain: Why and when. Best Pract Res Clin Rheumatol, 29(1):120–30. 26267006 10.1016/j.berh.2015.04.022 PMC4534717

[3] Lavie CJ Arena R Swift DL (2015). Exercise and the cardiovascular system: clinical science and cardiovascular outcomes. Circ Res, 117(2):207–19. 26139859 10.1161/CIRCRESAHA.117.305205 PMC4493772

[4] Conceição LS Neto MG do Amaral MA (2016). Effect of dance therapy on blood pressure and exercise capacity of individuals with hypertension: A systematic review and meta-analysis. Int J Cardiol, 220:553–7. 27390986 10.1016/j.ijcard.2016.06.182

[5] Lauche R Langhorst J Lee MS (2016). A systematic review and meta-analysis on the effects of yoga on weight-related outcomes. Prev Med, 87:213–32. 27058944 10.1016/j.ypmed.2016.03.013

[6] West J Otte C Geher K (2004). Effects of Hatha yoga and African dance on perceived stress, affect, and salivary cortisol. Ann Behav Med, 28(2):114–8. 15454358 10.1207/s15324796abm2802_6

[7] Li X Wang H Yang Y (2015). Effect of Height on motor coordination in college students participating in a dancesport program. Med Probl Perform Art, 30(1):20–25. 25743602 10.21091/mppa.2015.1003

[8] Yeung A Kiat H Denniss AR (2014). Randomised controlled trial of a 12 week yoga intervention on intervention on negative affective states, cardiovascular and cognitive function in post-cardiac rehabilitation patients. BMC Complement Altern Med, 14:411. 25342209 10.1186/1472-6882-14-411 PMC4218996

[9] Heyward VH Gibson AL (2014). Advanced fitness assessment and exercise prescription. 7th Ed eBook. Human Kinetics.

